# Level of women-friendly care provision among mothers in immediate post-partum period at public hospitals of Southeast Ethiopia: a cross-sectional study

**DOI:** 10.1186/s12905-022-02061-w

**Published:** 2022-11-24

**Authors:** Chala Kene, Yohannes Tekalegn, Diriba Dibaba, Mujib Abdella, Genet Fikadu, Daniel Atlaw, Degefa Gomora, Girma Geta, Kenbon Seyoum, Sintayehu Hailu, Neway Ejigu, Biniyam Sahiledengle, Alelign Tasew

**Affiliations:** 1 Midwifery Department, School of Health Science, Madda Walabu University, Bale-Goba, Ethiopia; 2 Public Health Department, School of Health Science, Madda Walabu University, Bale-Goba, Ethiopia; 3 Department of Human Anatomy, School of Medicine, Madda Walabu University, Bale-Goba, Ethiopia

**Keywords:** Women-friendly care, Respectful maternity care, Post-partum care

## Abstract

**Background:**

The women-friendly care approach focuses on women’s rights to have access to quality care for themselves as individuals, as mothers, and for their infants. However, access to quality health services is not guaranteed for many women, particularly in low and middle-income countries. Hence, this study aimed to assess the level of women-friendly care provision and associated factors among mothers in the immediate post-partum period at public hospitals of Bale Zone, Southeast Ethiopia 2021.

**Methods:**

An institutional-based cross-sectional survey was employed among mothers in the immediate post-partum period in public hospitals of Bale Zone from March 1–30, 2021. A total of 363 mothers were recruited by systematic random sampling technique in this study. Data was collected through pre-tested structured questionnaires. A 21-verified questionnaire was used to measure the outcome variable. The data were entered into Epi Data version 4.6.2.0 and exported to the statistical package of social science version 26.0 for analysis. A variable with a *P* value of less than 0.25 in the bi-variable binary logistic regression model was transferred to a multivariable binary logistics regression model. Hosmer and Lemeshow’s goodness of fit model was checked. Adjusted odds ratio with 95% confidence intervals were used to estimate the strength of association between the outcome variable and independent variables. A *p*-value less than 0.05 was considered as significantly associated.

**Results:**

The level of women-friendly care provision among mothers in immediate post-partum at public hospitals of Bale Zone was found to be 61% [95% confidence interval (55.73–66.04)]. Being prim para mother [Adjusted odds ratio = 1.88(1.07–3.33)], having planned pregnancy [Adjusted odds ratio = 1.94(1.04–3.63)] and staying at a health facility after delivery [Adjusted odds ratio = 4.8(1.71–13.39)] were found to be statistically significant predictors of level of women-friendly care provision.

**Conclusion:**

The women-friendly care provision among mothers in the immediate post-partum period in this study area was found to be low against most of the pre-existing findings. Strong counseling on planned pregnancy and staying at a health facility after delivery is recommended.

## Background

Every year more than 289,000 women die during pregnancy or childbirth due to severe maternal complications over the globe due to different reasons including substandard care provision in health facilities [1]. Evidence report that the less change in maternal mortality trend and fatality rates documented for obstetric complications indicate the lack of improvement in the quality of maternal health care provision [2].The main barriers that contribute to the low quality of women's health services include the lack of compliance of services with defined standards, the shortage of supplies, infrastructure problems, deficiency in detection and management of complications or emergency cases, and poor client-provider interaction [3].

According to Windau-Melmer, service providers must ensure that every woman seeking care is a person of value and has the right to be treated with respect and consideration [4]. However, still, access to quality health services is not guaranteed for many women, particularly in low and middle-income countries (LMICs) [5]. As a result, women were suffered from significant mistreatment in health facilities. Study in Ghana said, 83% of women suffered from at least one form of mistreatment during facility childbirth [6]. Other quantitative studies reported a prevalence of mistreatment in facility-based childbirth ranging from 15 to 98%, with most studies measuring mistreatment prevalence ranging from 12 to 20% in Tanzania, Kenya, and India [7]. Because of services paid, little attention to their needs, and showed little sensitivity towards local culture, women in Peru were reluctant to utilize Emergency Obstetrics Care(EmOC) facilities [8].

Ethiopia has one of the highest maternal mortality ratios (MMR) that is 401 maternal deaths per 100,000 live births in 2019 [9] and studies showed that the lack of women-friendly care during facility-based childbirth is continuing as a problem. For instance, in Ethiopian public health facilities, at least one form of mistreatment of women was committed in 36% of the observation in which 38% in health centers and 32% in hospitals [10]. Specifically, the study conducted in Jimma, Bahir Dar, Arba Minch revealed that (29%), (43%) and (98.9%) of women were did not receive women-friendly care services during facility-based childbirth respectively [11–13].

Poorly perceived quality of women-friendly care in facility-based childbirth often acts as a deterrent to current and/or future utilization of facility-based childbirth services. A recent assessment in Ethiopia showed that from 103 of the women interviewed, 85% of them reported that their service experience influenced their decision on where to deliver in the future [14]. Providing good-quality care is one of the most effective ways of ensuring that maternal health services are used and can be achieved through improving the women-friendly care health services which focuses on the rights of women to have access to quality care for themselves as individuals and as mothers, and for their infants [3].

It is a key intervention to improve the quality and bring unreached women to health facilities for maternity care services, although, full attention was not given to the level of women-friendly care provision and factors associated. As far as we know, in the study area, there is lack of evidences on the level of women-friendly care provision at public health facilities. Hence, this study aimed to assess the level of women-friendly care provision and its associated factors among mothers in the immediate post-partum period at public hospitals of southeast Ethiopia.

## Methods

### Study setting, design, and period

An institutional-based cross-sectional study was conducted at public hospitals of Bale Zone, from March 1–30, 2021. Bale zone is located in Southeast Ethiopia. Robe, the Zone city, is located 435 km far from the capital town of Ethiopia; Addis Ababa. Based on the report of Bale Zonal health department, the Zone has a total population of 1,269,951 from which women of childbearing age is around 281,040. The estimated number of pregnant women in the zone is around 44,067. Currently, the zone has five hospitals namely, Goba Referral Hospital, Delo Mena General Hospital, Madda Walabu General Hospital, Goro Hospital, and Robe General Hospital which is under robe town administration. Among five public hospitals, only Goba Referral Hospital, Delo Mena General Hospital, and Madda Walabu General Hospital were providing labor and delivery services during the data collection period. Robe General Hospital served as the center for COVID19 and Goro General Hospital was non-functional (Bale zone health department report, 2021).

### Study population

All mothers who gave birth in public hospitals of Bale Zone and in the immediate post-partum period were the source population, whereas randomly selected mothers who were in the immediate post-partum period were the study population. In this study, mothers with severe complications of birth were excluded.

### Sample size determination

The sample size was calculated using the single population proportion formula, assuming 71% proportion of women-friendly care provision from an institutional-based cross-sectional study conducted in public Hospitals of Jimma Zone, Ethiopia [11], 95% confidence interval (CI), and 5% margin of error. *n* = (1.96)2 0.71(0.29)/ (0.05)2 = 316. After adding a 15% non-response rate, the final sample size was found to be 363.

### Sampling procedure

According to the report from the zonal health department, the total number of delivery for the last three months was 1990, 289, and 137 in Goba Referral Hospital, Delo Mena General Hospital, and Madda Walabu General Hospital respectively. The monthly average number of delivery for these three hospitals was 663, 96, and 46 respectively. Further allocation of the sample was done for these hospitals proportionally based on the average number of clients who have received childbirth services in the last three months. So, the final sample size was 299, 43, and 21 for respective hospitals. Individual participants in each of the hospitals were selected by systematic sampling technique every K of 2 using a list of a mother who gave birth and recorded on postnatal registration book for the last three months as the sampling frame.

### Data collection tool and data collection procedure

A structured questionnaire which was adapted from different works of literature and consists of 36 questions and 21 verification criteria [3, 11, 15] is used in this study to assess the level of women-friendly care provision. It was made up of four sections, the first section was used to assess socio-demographic characteristics of the mother with 13 items, the second section was used to assess obstetric characteristics of participants with 13 items, the third section was used to assess facilities services use and providers related characteristics with 10 items, the fourth section was used to measure the level of women-friendly care provision experienced by mothers during facility childbirth which measured by 21 verified questions. The value 1 is given for those who answered ‘yes’ and 0 for those who answered ‘no’ and coded. The opposite questions were recoded. Then values were computed and a score of at least mean and above was taken as a good level of women-friendly care provision. In this study, four data collectors and four supervisors were assigned to facilitate the data collection. Data were collected during the immediate post-partum period in post-natal ward through interviews administered questions prepared by local language.

### Study variables, outcome variable, and operational definitions

Socio-demographic, provider, facility-related, and obstetrics variables were study variables whereas the level of women-friendly care provision was outcome variable.

A series of 21 questions related to the level of women-friendly care provision were asked. Study participants who scored at least score of 50% towards women-friendly care provision questions were categorized as getting a "good level of women-friendly provision" and else were categorized as getting poor women-friendly care provision.

Severe birth complications were complications like postpartum convulsion due to eclampsia and severe birth canal injury secondary to instrumental deliveries.

### Data quality assurance

An adopted questionnaire prepared in English was translated to Afan Oromo and then translated back to English by another language expert to check and maintain its consistency. Pretest was done to improve clarity, understandability, and simplicity of the messages of the tools before actual data collection. After pretest, modification on minutes needed for interviewing was corrected from 5–10 min to 10–15 min and some words was re-write as per suggestions from data collectors to make it simply understandable and clear tool which helps to meet the objective of the study. Data collectors and supervisors were trained for two days on the objective, method, sampling technique, ethical issues, data collection instrument, and data collection procedure before data collection. All of the questionnaires were checked for completeness and accuracy during and after the period of data collection.

### Data processing and analysis

The data were entered into Epi Data 4.6.2.0 and were coded. After that, it was exported to SPSS version 26.0 and cleaned before analysis. Descriptive statistics were calculated for the variables. Statistical significance and strength of the association between independent variables and outcome variables were analyzed by using the logistic regression model. A variable with a *P* value of less than 0.25 in the bi-variable binary logistic regression model was transferred to a multivariable binary logistics regression model to adjust the confounder’s effects. The enter method was used to run the model. Hosmer and Lemeshow’s goodness of fit model was checked and the data fitted the model well (*p* = 0.92). Crude and adjusted odds ratios with their 95% confidence intervals were calculated. Adjusted odds ratio with 95% confidence intervals were used to estimate the strength of association between the outcome variable and independent variables. A *p*-value less than 0.05 was considered as significantly associated. Finally, the result of the study was presented using tables, chart, and texts based on the data obtained.

## Result

In this study a total of 363 study participants participated, yielding a response rate of 98.9%, whereas the remaining 1.1% non-response rate was due to incomplete data not due to the sensitivity of the questionnaires.

### Socio-demographic characteristics of the study participants

Most of the participants 281(78.3%) were Oromo in ethnicity followed by Amhara 64(17.8%). The mean age of participants was 28.34 years with a standard deviation of ± 5.51SD and a range from age of 17 to 42 years. The majority 334(93.0%) of the participants were married and 175(48.3%) of them were Muslim followers whereas 11(3.1%) were Waqeffatta. Of total the participants, 92(25.6%) had a primary level of education while 244(68.0%) participants’ means of transportation to health facility was public transport which took 30 min in 173(48.2%) of the participants (Table 1).

**Table 1:** Socio-demographic characteristics of mothers in the immediate post-partum period at public hospitals of Bale Zone, Southeast Ethiopia 2021(*n* = 359)

Variables	Frequency	%
Age		
< = 34	300	83.6
> = 35	59	16.4
Ethnicity		
Oromo	281	78.3
Amhara	64	17.8
Others^1^	14	3.9
Religion		
Muslim	175	48.7
Orthodox	122	34.0
Protestant	51	14.2
Waqeffatta	11	3.1
Residence		
Rural	161	44.8
Urban	198	55.2
Who else lives with you in your household		
Lives alone	20	5.6
Husband/partner	285	79.4
Children	16	4.5
One or more parent	28	7.8
One or more parent-in-law	7	1.9
Other relatives	3	.8
Mother’s level of education		
No formal education	88	24.5
Primary education	92	25.6
Secondary education	75	20.9
College/university	104	29.0
Partner’s level of education		
No formal education	76	21.2
Primary education	46	12.8
Secondary education	65	18.1
College/university	159	44.3
Mother’s occupation		
Housewife	187	52.1
Merchant	77	21.4
Government Employee	80	22.3
Non-government employee	15	4.2
Family monthly income in Ethiopian birr		
< = 1999	115	32.0
> = 2000	244	68.0
Marital status		
Single	13	3.6
Married	334	93.0
Divorced	6	1.7
Widowed	6	1.7
Main means of transport to get health institutions		
Walk	52	14.5
Public transport(bus, taxi)	244	8.0
Private vehicle	12	3.3
Private motor cycle	26	7.2
Ambulance	25	7
How long does it take to get health institutions from your home		
1–30 min	173	48.2
30-1 h	107	29.8
1-2 h	46	12.8
> 2 h	31	8.6
Don't know	2	.6

Key: Other^1^, Sidama, Woloyitta, Tigre, Sumale

### Obstetric history of the participants

Regarding deliveries, 279(77.7%) participants had more than one number of deliveries, and 285(79.4%) last pregnancy was planned. Among participants who had ANC follow-up, only 155(53.7%) had four and more times of follow up while 38(10.6%) participants did not have ANC follow up at all. Considering the type of mode of delivery 208(57.9%) participants gave birth through vaginal delivery followed by cesarean Sect. 80(22.3%). Of the total respondents, 267(74.4%) received postnatal care in the immediate postpartum period. During the last delivery, 116(32.3%) participants had experienced birth complications in which 44(12.3%) developed postpartum hemorrhage and 9(2.5%) developed inversion of the uterus (Table 2).

**Table 2:** Obstetric characteristics of mothers in the immediate post-partum at public hospitals of Bale Zone, Southeast Ethiopia 2021 (*n* = 359)

Variables	Frequency	%
Parity		
1	80	22.3
> = 2	279	77.7
Did your last pregnancy was planned?		
Yes	285	79.4
No	74	20.6
Did you have Antenatal care follow-up during the pregnancy?		
Yes	321	89.4
No	38	10.6
How many times did you have ANC follow-up during pregnancy?		
1–3	116	46.3
> = 4	155	53.7
Where did you give birth to a recent baby		
Public health center	27	7.5
Public hospital	328	91.4
Private clinic	2	.6
Home	2	.6
Type of last delivery		
Normal delivery		
Cesarean delivery	208	57.9
Vacuum extraction/forceps delivery	80	22.3
Delivery by episiotomy	27	7.5
Experience complication during last delivery		
Yes	116	32.3
No	243	67.7
Type of complication		
Postpartum Hemorrhage	44	12.3
Retained placenta	19	5.3
Injury on birth canal	16	4.5
Inversion of uterus	9	2.5
Cesarean section secondary to prolonged labor	28	7.8
Did you received postnatal care in immediate postpartum period		
Yes	267	74.4
No	92	25.6
Did you stay in health facility after delivery		
Yes	339	94.4
No	20	5.6
For how many days did you stay in health facility after delivery		
6 h	162	45.1
One day	94	26.2
Two days	52	14.5
More than 2 days to one week	23	6.4
More than a week	8	2.2

### Facility services use and providers related characters

Most of the participants 204 (56.8%) reported average waiting time to see a provider during a visit was less than 30 min. Of the total respondents, 19(5.3%) paid money during the care provision even if maternity care was free of charge in Ethiopia. Regarding the sex of the main providers assisting the participants during delivery, 234(65.2%) participants were assisted by a midwife, and the main 232 (64.5%) sex of care provider was male. And among the participants, 28(7.8%) were treated badly in which 12(3.3%) of them were treated badly by the doctor followed by midwife 10(2.8%) and the main type of abuse reported by study participants was psychological abuse which was shouting at them in 12(3.3%) participants (Table 3).

**Table 3:** Facility and providers related services for mothers during visit of public hospitals in Bale Zone, Southeast Ethiopia 2021 (*n* = 359)

Variables	Frequency	%
Average waiting time to see provider during visit		
Less than 30 min	204	56.8
30 min to 1 h	114	31.7
1 and half hour to 2 h	29	8.1
More than 3 h	12	3.4
Did you have to pay for services?		
Yes	19	5.3
No	340	94.7
Who assisted you with delivery of the baby		
Doctor	99	27.6
Midwife	234	65.2
Both	20	5.6
Other^2^	26	1.8
Sex of main providers assisting your delivery		
Male	232	64.6
Female	127	35.4
Anyone who treated you badly in some way		
Yes	28	7.8
No	331	92.2
Who treated you badly		
Midwifery	10	2.8
Doctor	12	3.3
Porter	2	.6
Cleaner	5	1.4
What they did to you		
Shouted/scolded	12	3.3
Examined roughly	3	.8
Didn't come when called	6	1.7
Physically hit, slapped, pushed pinched or otherwise beat you	2	.6
Verbally(insulting) abuse during labor or delivery	5	1.4

Key: Other^2^, Midwife + Emergency surgeon of obstetrics (ESO)

### Provision of women-friendly care

A total of 21 questions were used to measure the provision of women-friendly care among the participants. The value 1 is given for those who answered ‘yes’ and 0 for those who answered ‘no’ and coded. The opposite questions were recoded. The sum of these questions was calculated to range from 0 to 21 with a mean women-friendly care provision of 12.24 and SD of 3.68.

As indicated in the table below, 337(93.7%), 322(89.7%), 320(89.1%) and 257(71.6%) respondents answered ‘yes’ to the question that asked if received care from the skilled birth attendant, if health facility provides them with the continuous availability of basic supplies, equipment or drugs, if they satisfied with waiting time in a health facility during health services provisions and if the language spoken by the health worker was easy to understand respectively. In another case, 217(60.3%), 272(75.8%) and 331(92.2%) respondents answered “no” to the questions if health facility provides them access to the ambulance to reach health institutions, if health care facility provides them with clean bathroom, toilet & water and if health care facility charge for services, drugs, and supplies respectively (Table 4).

**Table 4:** Women-friendly services experienced at public hospitals in Bale Zone, Southeast Ethiopia 2021 (*n* = 359)

	Variables	Yes	No
		Freq (%)	Freq (%)
**A**	**Accessibility of health care services**		
	Health facilities provided you with access to ambulance to reach this health institution	142(38.6)	217(60.4)
	Satisfied with waiting time in a health facility during your health services provisions	320(89.1)	39(10.9)
**B**	**Availability of health care services**		
	Health facility provided you with the continuous availability of basic supplies, equipment, or drugs	322(89.7)	37(10.3)
	Received care from the skilled birth attendants	337(93.9)	22(6.1)
**C**	**Culturally acceptability of health care services**		
	Satisfied with the sex of your birth attendant	309(86.1)	50(13.9)
	Health workers respect you regardless of your cultural norms, religion, and believe throughout your stay in this health facility	294(81.9)	65(18.1)
**D**	**Empowerment and satisfaction of mothers**		
	Language spoken by the health worker was easy to understand	257(71.6)	102(28.4)
	The health worker greeted and introduced him/her to you when you arrived	266(74.1)	93(25.9)
	Treated you with dignity /confidentiality	261(72.7)	98(27.3)
	Informed and allowed you to have a companion of your choice during labor	148(41.2)	211(58.8)
	Obtain consent or permission from you prior to any procedures	216(60.2)	143(39.8)
	Providers explained to you what is being done and what to expect throughout labor and birth and during any procedures or examinations	216(60.2)	143(39.8)
	Encourages you to ask questions and respond with politeness and truthfulness	209(58.2)	150(41.8)
	Called you or referred to you by your name and not by others (e.g. bed number or health problem)	261(72.7)	98(27.3)
	Health care providers informed you of different delivery positions and allowed you to adopt the delivery position of your choice	195(54.3)	164(45.7)
**E**	**Affordability of health care facility**		
	Health care facilities charge for services, drugs and supplies	28(7.8)	331(92.2)
**F**	**Infrastructures of the facility**		
	Health care facilities provide you with clean bathroom, toilet & water	87(24.2)	272(75.8)
	Maternity ward kept clean(beds, floors,windows, walls & linen)	183(51.0)	176(49.0)
	Use linen, curtains or screens to ensure your privacy during labor and delivery	194(54.0)	165(46.0)
	Keep examination room separately	78(21.7)	281(78.3)
	Health facility provide you with adequate waiting space	73(20.3)	286(79.7)

**Fig. 1:**
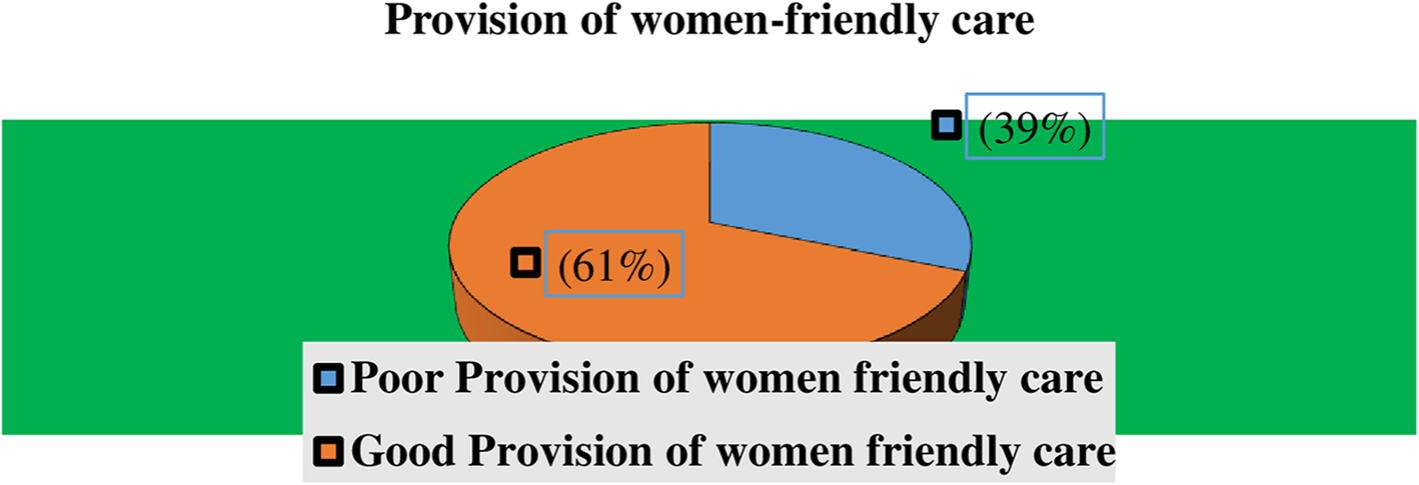
Level of women-friendly care provision among mothers in the immediate post- partum period at public hospitals of Bale Zone, Southeast Ethiopia 2021

As the below figure shows, more than half 219(61%) of participants got the good provision of women-friendly care which is greater or equal to the mean value, and 140(39%) participants got the poor provision of women-friendly care (Fig. 1).

### Factors associated with provision of women-friendly care

In this study, the association of different background factors of the study participants with a level of women-friendly care provision was investigated using bivariate and multivariable binary logistic regression analysis. On bivariate binary logistic regression analysis; residence, mother’s level of education, family monthly income, parity, last pregnancy planned, stay at a health facility after delivery, and paid for services were found to be statistically significant.

Variables found statistically significant on bivariate binary logistic regression analysis and had predetermined *p*-value less than 0.25 were entered into multivariable binary logistic regression. During multivariable binary logistic regression; parity, last pregnancy planned, and stay at a health facility after delivery were found to be statistically significant predictors of women-friendly care provision.

Primi para mother had almost 2 times more likely to have good women friendly care provision than multi mother [AOR = 1.88(1.07–3.33)]. In addition, participants who did plan their last pregnancy were 2 times more likely to have good women friendly care provision than those who did not plan [AOR = 1.94(1.04–3.63)], whereas participants who stayed at a health facility after delivery were found to be almost 5 times more likely to have good women friendly care provision than those who did not stay at a health facility after delivery [AOR = 4.8(1.71–13.39)] (Table 5).

**Table 5:** Factors associated with the provision of women-friendly care among mothers at the immediate post-partum period at public hospitals of Bale Zone, Southeast Ethiopia 2021 (*n* = 359)

Variables	Provision of women-friendly care		COR(95%CI)	AOR(95%CI)	*P*-value
	Poor N (%)	Good N (%)			
Residence					
Rural	54(15)	107(30)	1.52(0.99–2.34)	1.43(0.83–2.48)	0.206
Urban	86(24)	112(31)	1.00	1.00	
Mother’s level of education					
No formal education	30(8)	58(16)	1.42(0.79–2.55)	1.87(0.83–4.22)	0.134
Primary education	30(8)	62(18)	1.52(0.85–2.72)	1.68(0.85–3.33)	0.139
Secondary education	36(10)	39(11)	0.79(0.44–1.44)	0.85(0.46–1.58)	0.600
Collage/university	44(12)	60(17)	1.00	1.00	
Family monthly income					
< = 1999	39(11)	76(21)	1.38(0.87–2.19)	1.09(0.58–2.06)	0.795
> = 2000	101(28)	143(40)	1.00	1.00	
Parity					
1	24(7)	56(16)	1.66(0.97–2.83)	1.88(1.07–3.33)	0.029*
> = 2	116(32)	163(45)	1.00	1.00	
Last pregnancy planned					
Yes	105(29)	180(50)	1.54(0.92–2.58)	1.94(1.04–3.63)	0.039*
No	35(10)	39(11)	1.00	1.00	
Stay at health facility after delivery					
Yes	126(35)	213(59)	3.94(1.48–10.53)	4.8(1.71–13.39)	0.003*
No	14(4)	6(2)	1.00		
Paid for service					
Yes	11(3)	8(2)	0.45(0.17–1.13)	0.43(0.16–1.17)	0.097
No	129(40)	211(59)	1.00		

*Abbreviations*: *COR* Crude Odds Ratio, *AOR* Adjusted Odds Ratio, *CI* Confidence Interval

^*^ Significant at *p* < 0.05 (Adjusted)

## Discussion

This study assessed women-friendly care provision and associated factors among mothers in the immediate post-partum period at public hospitals of southeast Ethiopia. The finding showed that the women-friendly care provision was (61%) [95%CI (55.73–66.04)] in the study area. The finding of this study was in line with a study conducted in Tanzania 63% [16], however, higher than related study conducted in Western (25.2%), Eastern (38.4%) Ethiopia [17, 18], and Bahir Dar (57%) [12], but lower than the study conducted in Jimma (71%), Gamo Gofa (79.1%), Harar Hospitals (85%) and Amhara region hospital (88%) [11, 19–21].

The possible reason for the discrepancies may be due to adherence to health facilities with women-friendly care amenities and different characteristics of health care providers in these different health facilities which affect the care provision. In these different places, there may be different capacity building in training techniques for health care providers result in different levels of women-friendly care provision. In addition, the possible reason may be particularly participant’s level of understanding about the services, service quality, and perception of normalizing some mistreatments.

Evidence report that disrespect and abuse of women during maternity care are problems that have been masked by a veil of silence and they can expressively impact women’s readiness to seek life-saving maternity care [4]. According to this study, of the total participants (7.8%) were disrespected during facility-based childbirth. This finding was lower than studies conducted in Egypt [22], Bahir Dar [12], and Arba Minch, Ethiopia [13]. The difference may be due to differences in health policy regarding maternity care across the countries and the level of applying that policy in the right ways in different administrations of the countries. This finding shows that still significant number of mothers are disrespected during post-delivery services while they bring newborns into this world. This type of activity may hinder women from seeking maternity care. If not resolved the lack of women-friendly care in facility-based childbirth often acts as a deterrent to future utilization of facility-based childbirth services [14]. Therefore, attention should be given to delivering quality-based maternity services during the immediate post-partum period, the crucial period after labor pain.

As the study revealed prim para mothers had almost 2 times more likely to have good women-friendly care provision than multipara mothers. The possible reason may be primipara mothers may be strange for the environment and assume the care provided by health facility as it is perfect whereas multipara mothers knew the care provided by health facility in a good manner from the experience and graded the health facilities service from the experience. However, the result disagreed with the previous related study conducted [23].

In addition, participants who did plan their last pregnancy were 2 times more likely to have good women-friendly care provision than those who did not plan. Similarly, last pregnancy was associated with women-friendly care in the study conducted in Eastern part of Ethiopia [18]. The possible reason may be, as pregnancy intentional, wanted and planned; utilization of maternal health services increased that help the mothers to be familiarized with friendly care health services, with health care providers that reduced sense of mistreated and increase the women’s attitude to perceive the care as friendly and respectful.

Correspondingly, in this study participants who stayed at a health facility after delivery were found to be almost 5 times more likely to have good women-friendly care provision than those who did not stay at a health facility after delivery. The finding was agreed with the related study conducted at Harar Hospitals [20]. The probable reason may be as long as the women stayed at health facilities they may perceive that they got good women-friendly care provision. The other reason may be as they stayed at health facilities they may familiarized with the environment and request good services.

It can be concluded that good women-friendly care provision at the health facilities level has multidirectional benefits at large and is clinically important to decreasing maternal and neonatal mortality rates. It should be provided regardless of circumstances, because all women have the right of to be well-treated. Irrespective of the variables associated with good woman-friendly care, all women who give birth in a health facility should be treated equally. The finding implies that there is still substandard postpartum care in developing countries like Ethiopia. As a result, there is a need to improve immediate postpartum care at the clinical level in each health facility.

Even if the study follows scientific procedural approach carefully, however, it had the following limitations. The firs one is, since the data were collected in the health facility there may be social desirability bias and fear of reporting mistreatment. To decrease this bias, the participant’s privacy was kept through using screen as a shelter for the mothers during data collection. The second one is, since the data was collected in the immediate post-delivery period it was difficult for the women to respond to the questions. The third one is, since the nature of the study design was cross-sectional, the study may difficult to ascertain the causal relationship between the study variables.

## Conclusion

In general, compared to most of the other findings, the level of women-friendly care provision in public hospitals of Southeast Ethiopia was found to be low. Variables like parity, last pregnancy planned, and stay at a health facility after delivery were found to be statistically significant predictors of women-friendly care provision.

Since providing good women-friendly care is measure of quality care for health facilities, health care providers should be trained well to provide good women-friendly. Providing good women-friendly care is clinically important to increase health professional’s job satisfaction and helps to tackle maternal mortality rate.

The management of hospitals should work on better infrastructures, to provide better health care services and regularly evaluate the condition of women-friendly care services in the postnatal ward and should take correction measures.

Besides, that all supportive staff should be cooperative in providing good women-friendly care including cleaners and others. Furthermore, strong counseling on planned pregnancy and staying at health facilities after delivery is recommended. At the last, but not the least, researchers recommended working more in the areas by using a mixed study design.

## Data Availability

The data set analyzed during the current study are not publicly available due to consent for publication was not taken from the participants, but are available from the corresponding author on reasonable request.

## Abbreviations

ANC: Ante Natal Care
CEmOC: Comprehensive Emergency Obstetric care
MMR: Maternal Mortality Rate
SPSS: Statistical Package of Social Science
CI: Confidence interval
COR: Crude odds ratio
AOR: Adjusted odds ratio
SD: Standard Deviation
MWU: Madda Walabu University
LMICs: Low and middle-income countries
